# A Case of Acquired Methemoglobinemia Following Sentinel Lymph Node Dye With Patent Blue V

**DOI:** 10.7759/cureus.73897

**Published:** 2024-11-18

**Authors:** Gülay Esen, Melek Civi

**Affiliations:** 1 Department of Anesthesiology and Intensive Care, Sivas Gemerek State Hospital, Sivas, TUR; 2 Department of Anesthesiology and Intensive Care, Celal Bayar University Training and Research Hospital, Manisa, TUR

**Keywords:** breast cancer surgery, methemoglobinemia, methylene blue, patent blue v, sentinel lymph node biopsy

## Abstract

Methemoglobinemia is a rare but potentially life-threatening condition in which hemoglobin is oxidized, impairing the oxygen-carrying capacity. While congenital forms exist, acquired methemoglobinemia can occur in perioperative settings, especially following exposure to oxidizing agents such as dyes used in sentinel lymph node biopsy (SLNB). Patent Blue V, a synthetic aniline dye commonly used for SLNB, has been associated with rare but serious adverse effects, including methemoglobinemia.

We report a case of acquired methemoglobinemia in a 68-year-old female undergoing bilateral modified radical mastectomy with SLNB for breast carcinoma. The patient experienced a sudden and progressive decline in oxygen saturation (SpO2) following administration of 6 ml of 2.5% Patent Blue V. Despite supplemental oxygen therapy, hypoxia persisted, prompting arterial blood gas (ABG) analysis, which confirmed methemoglobinemia (9.2%). Immediate treatment with methylene blue (1 mg/kg) resulted in rapid clinical improvement, with the methemoglobin levels normalizing within eight hours.

This case highlights the importance of early recognition and treatment of acquired methemoglobinemia, particularly in a perioperative setting. Patent Blue V is associated with both hypersensitivity reactions and methemoglobinemia, and its interference with pulse oximetry readings can complicate intraoperative monitoring. Prompt administration of methylene blue remains the standard treatment, leading to favorable outcomes.

Clinicians should be vigilant regarding the possibility of methemoglobinemia when using dyes such as Patent Blue V during surgical procedures. Diagnostic tools, such as ABG or co-oximetry, are essential for accurate diagnosis, and early treatment with methylene blue can prevent serious complications.

## Introduction

Methemoglobinemia is a rare hematologic disorder characterized by an elevated concentration of methemoglobin, an oxidized form of hemoglobin, in which the iron component is in the ferric (Fe^3+^) state rather than the ferrous (Fe^2+^) state. In this state, methemoglobin is incapable of effectively binding and releasing oxygen, thereby impairing oxygen delivery to tissues and leading to hypoxia. Methemoglobinemia occurs when hemoglobin, the protein responsible for transporting oxygen in the blood, is altered to not release oxygen effectively to body tissues. This leads to hypoxia, where organs and tissues lack adequate oxygen. The condition can be congenital, due to genetic enzyme deficiencies, or more commonly acquired due to exposure to specific exogenous agents, such as oxidizing medications, chemicals, and dyes. Acquired methemoglobinemia is of particular concern in perioperative settings, where exposure to specific pharmacological agents and dyes may precipitate this condition [1,2].

Patent Blue V, a synthetic aniline dye, is frequently used in sentinel lymph node biopsy (SLNB) during oncological surgeries, such as breast cancer procedures. This technique facilitates the identification of sentinel lymph nodes by staining the lymphatic vessels, aiding in the precise excision of the primary nodes for pathological evaluation. Patent Blue V in SLNB has demonstrated substantial prognostic utility with low morbidity and is pivotal in accurate cancer staging and treatment planning [3]. SLNB is a surgical technique used in cancer procedures to identify the first lymph node (sentinel node) that a tumor's cells are likely to spread to. This helps stage cancer and plan treatment. Patent Blue V is a synthetic dye commonly used during SLNB to visually map the lymphatic system and identify the sentinel lymph node. However, despite its clinical benefits, the administration of Patent Blue V is not without risks. Documented adverse reactions range from mild cutaneous hypersensitivity to severe systemic reactions, including anaphylaxis and acquired methemoglobinemia [4-6]. The occurrence of methemoglobinemia following Patent Blue V exposure, although rare, warrants clinical vigilance because of its potential to precipitate rapid-onset hypoxemia that is refractory to supplemental oxygen therapy [7,8]. Patients may present with cyanosis, headaches, dizziness, and, in more severe cases, life-threatening complications such as arrhythmias, seizures, or coma. Furthermore, the presence of Patent Blue V can confound intraoperative monitoring as the dye interferes with pulse oximetry by artificially lowering oxygen saturation readings, thus complicating the detection of true hypoxemia [9-11].

Given these potential risks, heightened awareness and prompt identification of methemoglobinemia in the perioperative setting are imperative for anesthesiologists and surgical teams. The standard of care for suspected or confirmed methemoglobinemia includes the administration of methylene blue, which acts as a reducing agent to convert methemoglobin back to hemoglobin, in conjunction with supportive measures such as oxygen supplementation and intravenous hydration. In this report, we describe a case of methemoglobinemia induced by Patent Blue V during a bilateral modified radical mastectomy, underscoring the importance of early recognition and appropriate management to mitigate adverse outcomes.

## Case presentation

A 68-year-old female patient weighing 68 kg with a diagnosis of breast carcinoma was scheduled for a bilateral modified radical mastectomy with axillary lymph node dissection. The patient's medical history was significant for well-controlled hypertension. The patient had no history of alcohol or tobacco use, known allergies, or surgical interventions. Preoperative evaluations, including cardiovascular and respiratory system assessments, were unremarkable, with a Mallampati score of II. The routine preoperative laboratory workup was within normal limits. Intravenous access was secured, and preoperative analgesia was achieved through the bilateral erector spinae plane (ESP) block using a total of 50 mg bupivacaine diluted in 10 cc saline.

The patient was transferred to the operating room, where standard monitoring was initiated. This revealed the following baseline parameters: blood pressure, 118/71 mmHg, heart rate of 85 beats per minute, and 99% oxygen saturation (SpO_2_). Following pre-oxygenation, general anesthesia was induced with 2 mg of midazolam, 150 µg of fentanyl, and 150 mg of propofol. Hemodynamic monitoring was performed during the surgical procedure. The patient also received intravenous diphenhydramine (25 mg) for a potential allergic reaction, alongside other perioperative medications.

During sentinel lymph node biopsy, 6 ml of 2.5% Patent Blue V dye was injected for lymphatic mapping. Approximately 10-15 minutes after administering 6 ml of 2.5% Patent Blue V dye for lymphatic mapping, the patient experienced a gradual decrease in oxygen saturation, falling to 89-90%, despite receiving supplemental oxygen. Anesthesia equipment, including the ventilator and breathing circuits, were inspected, and no faults were detected. Oxygen supplementation was increased to 100%, and an arterial blood gas (ABG) sample was obtained.

 Initial measures to improve oxygenation, including ensuring correct endotracheal tube placement and equipment functionality, were unremarkable. Persistent hypoxia prompted further investigation through ABG analysis, which is crucial because pulse oximetry readings could be falsely low due to the dye's interference. ABG analysis revealed methemoglobinemia (9.2%) as the underlying cause of hypoxia with elevated methemoglobin levels. Treatment was promptly initiated with methylene blue at a dose of 1 mg/kg, administered intravenously over 30 min, in conjunction with intravenous fluid therapy. Despite the transient oxygen desaturation, the surgical procedure proceeded without further complications. The patient was extubated successfully after surgery and transferred to the intensive care unit (ICU) for postoperative management.

Postoperative management

Upon admission to the ICU, the patient’s vital signs were stable: blood pressure, 110/70 mmHg; heart rate, 66 beats/min; and oxygen saturation (SpO2), 99%. The patient continued to receive methylene blue at a dose of 1 mg/kg intravenously every four hours for a total of three doses. The administration was carefully monitored, and the treatment was adjusted based on the patient's response. Regular ABG analyses showed a steady decline in methemoglobin levels. Oxygen supplementation was continued with high-flow oxygen therapy (FiO_2_ of 50%, 20 L/min) until methemoglobin levels normalized. Serial ABG analyses and the results were performed regularly (Table 1).

**Table 1:** Arterial blood gas (parameters and methemoglobin levels post-methylene blue treatment in a patient with methemoglobinemia). PO_2_: partial pressure of oxygen, PCO_2_: partial pressure of carbon dioxide, SO_2_: oxygen saturation level, MetHb: methemoglobin, FCOHb: carboxyhemoglobin.

Time (Post-treatment)	pH	PO_2_ (mmHg)	PCO_2_ (mmHg)	SO_2_ (%)	MetHb (%)	FCOHb (%)
Initial (Pre-treatment)	7.33	179.3	46.7	94	9.2	1.4
1 Hour	7.41	446.6	31.1	98.8	8.2	1.2
3 Hours	7.30	350	36.1	98	7.7	0.6
6 Hours	7.41	446.6	31.1	98.8	4.2	0.4
8 Hours	7.42	428.5	27.2	98.5	2.6	0.2
10 Hours	7.42	428.5	27.2	98.5	0.9	0.2

The patient's condition improved steadily, and no further desaturation episodes were noted during the postoperative period. This case underscores the importance of considering acquired methemoglobinemia in patients presenting with unexplained hypoxia after the use of dyes like Patent Blue V, particularly if symptoms are unresponsive to conventional oxygen therapy. Prompt diagnosis via ABG and timely treatment with methylene blue are crucial for rapid recovery.

## Discussion

This case highlights an instance of acquired methemoglobinemia following the use of Patent Blue V during bilateral modified radical mastectomy for breast carcinoma. A 68-year-old female with no prior significant allergic history developed a sudden and progressive drop in SpO2 after the injection of Patent Blue V for SLNB. Despite supplemental oxygen therapy, the patient's hypoxia persisted, prompting further investigation and diagnosis of methemoglobinemia by ABG analysis. Immediate treatment with methylene blue resulted in a favorable outcome, with the patient's condition stabilizing postoperatively.

Several case reports in the literature, such as those by Azevedo et al. and Korbi et al., describe similar occurrences of allergic reactions and methemoglobinemia following Patent Blue V administration [4,5]. These cases, as in the case presented here, require prompt intervention with methylene blue, the standard treatment for methemoglobinemia. Methylene blue works by reducing methemoglobin to functional hemoglobin, thereby restoring oxygen-carrying capacity. In the current case, the patient received 1 mg/kg of methylene blue intravenously, which successfully normalized the methemoglobin levels and resolved hypoxia over several hours.

The resolution of methemoglobinemia in this case mirrors the findings in the literature, where methemoglobin levels typically decline within 4-6 hours post-treatment with methylene blue. Studies by Yüksel et al. and Hueter et al. have similarly reported a rapid decrease in methemoglobin levels following treatment, with most patients returning to normal oxygenation within 8-10 hours [7,8]. The patient, in this case demonstrated a steady decline in methemoglobin levels, with near-complete resolution by the 8-hour mark, consistent with the pharmacokinetics of methylene blue, which has a half-life of 1-2 hours.

Prompt recognition and management of methemoglobinemia are critical in preventing serious complications such as arrhythmias, seizures, and, in severe cases, coma. Although methemoglobinemia is a rare complication of Patent Blue V administration, clinicians should be vigilant when using this dye, particularly for high-risk patients. The refractory nature of hypoxia despite supplemental oxygen, as observed in this case, is a hallmark of methemoglobinemia and should prompt further diagnostic testing using ABG or co-oximetry.

The interference of Patent Blue V with pulse oximetry is well-documented in the literature [7,9]. This can significantly complicate intraoperative monitoring as pulse oximeters may not accurately reflect the patient’s true oxygenation status. In the current case, despite the patient’s SpO2 dropping to 89-90%, hypoxemia was primarily due to elevated methemoglobin levels rather than failure of oxygen delivery, underscoring the importance of using co-oximetry or ABG analysis in such situations.

## Conclusions

Following the administration of Patent Blue V during SLNB, this acquired methemoglobinemia contributes to the growing body of literature on the potential adverse effects of aniline dyes in a perioperative setting. Although rare, methemoglobinemia can lead to significant morbidity if not promptly recognized and treated. Clinicians should know the risks associated with dyes, such as Patent Blue V, and be prepared to utilize diagnostic tools such as co-oximetry and ABG to confirm the diagnosis. Methylene blue remains the gold standard for treatment, with rapid clinical improvement typically observed following its administration. Clinicians should remain vigilant for potential complications such as methemoglobinemia when using dyes like Patent Blue V. Diagnostic tools like ABG and co-oximetry should be considered in cases of unexplained hypoxia, and alternative dyes may be appropriate for patients with potential risk factors. Future studies should focus on identifying patients at a higher risk for dye-induced methemoglobinemia and explore alternative dyes with safer profiles for use in SLNB.
